# Assessment of the General Nutrition Knowledge of Students from the University of Novi Sad (Vojvodina, Serbia)

**DOI:** 10.3390/nu16223918

**Published:** 2024-11-16

**Authors:** Miloš Ilić, Danica Ilibašić, Huiwen Pang, Tomislav Vlaški, Jelena Jovičić-Bata, Maja Grujičić, Budimka Novaković

**Affiliations:** 1 Department of Pharmacy, Faculty of Medicine, University of Novi Sad, Hajduk Veljkova 3, 21000 Novi Sad, Serbia; jelena.jovicic-bata@mf.uns.ac.rs (J.J.-B.); budimka.novakovic@mf.uns.ac.rs (B.N.); 2 Faculty of Medicine, University of Novi Sad, Hajduk Veljkova 3, 21000 Novi Sad, Serbia; ilibasicdanica@gmail.com; 3 Australian Institute for Bioengineering and Nanotechnology, The University of Queensland, Brisbane, QLD 4072, Australia; 4 School of Biomedical Sciences, Faculty of Medicine, The University of Queensland, Brisbane, QLD 4072, Australia; 5 Medical Faculty Heidelberg, University of Heidelberg, Im Neuenheimer Feld 672, 69120 Heidelberg, Germany; tomislav.vlaski@dkfz-heidelberg.de; 6 Department of General Education Subjects, Faculty of Medicine, University of Novi Sad, Hajduk Veljkova 3, 21000 Novi Sad, Serbia; maja.grujicic@mf.uns.ac.rs

**Keywords:** nutrition knowledge, diet, nutrition, healthy eating habits, students, young adults, public health, Serbia, South-East Europe

## Abstract

**Background:** Healthy nutrition is necessary for a good quality of life and reduction in the risk of developing diseases. Research indicates that students do not usually have healthy eating habits. Knowledge about nutrition, dietary guidelines, food groups and the nutrients they contain, the selection and adequate preparation of food, and the health consequences of unhealthy nutrition can influence the eating habits of students. Until recently, no research had been conducted on university-level students’ nutrition knowledge in Serbia. The aim of this study was to determine the nutrition knowledge of students from the University of Novi Sad (Vojvodina, Serbia) in relation to gender, year of study, academic field of study, and nutritional status. **Methods:** A cross-sectional study was conducted in the period from December 2021 to July 2022, on 898 students (72.9% female and 27.1% male, with an average age of 22.87 ± 3.092). An online survey questionnaire was used as the research instrument. An assessment of nutrition knowledge was performed using the General Nutrition Knowledge Questionnaire (GNKQ) for the following domains: dietary recommendations; food groups and the nutrients they contain; healthy food choices; and diet, disease and weight associations. **Results:** Significant differences in students’ assessed knowledge levels were determined across various domains of healthy nutrition. The highest level of nutrition knowledge was in healthy food choices, and the lowest was in understanding food groups and nutrient composition. The total determined knowledge about healthy nutrition correlated most with self-assessed knowledge of food groups, and least with knowledge of dietary recommendations. Female gender and higher years of study were associated with higher nutrition knowledge. Students from the Faculty of Medicine had the highest nutrition knowledge, followed by students from the Faculty of Technology and the Faculty of Science, compared to students from other faculties. **Conclusions:** Our findings highlight the need for targeted interventions aimed at improving students’ understanding of specific nutritional concepts, ultimately empowering them to make informed dietary decisions for lifelong health and wellbeing.

## 1. Introduction

Healthy nutrition is necessary for the physiological growth, development, and maintenance of physical, cognitive, immunological, skeletal-muscular, and other functions of the body. A balanced energy intake and a sufficient intake of nutrients and regulatory substances maintain body weight and reduce the risk of non-communicable diseases (NCDs) [1,2]. The high prevalence of NCDs poses a significant public health challenge globally, which is heavily influenced by dietary practices. Unhealthy nutritional habits, characterized by low consumption of raw fruits, vegetables, and whole grains and high sodium intake, have been identified as a primary contributor to the global burden of disease, accounting for 7.9 million premature deaths and 187.7 million disability-adjusted life years (DALYs) in 2019 [3,4].

Starting a university education can represent a critical moment in the life of a young adult, when, due to higher faculty obligations and the influence of a new social environment, new patterns of nutritional behavior can occur [5,6]. Research has shown that, students do not usually have healthy eating habits. Common dietary patterns among students include irregular meals, skipping breakfast, preference for highly processed, energy-dense foods that contain a high share of saturated fats and trans-fatty acids, added sugar, and salt, and reduced consumption of fruits, vegetables, and whole grains. More than half of the student population consume “fast food” every day because of its easy availability, taste, and low cost [5,7,8,9].

Unhealthy life habits (such as unhealthy nutrition, a low level of physical activity, alcohol abuse, and cigarette smoking) in youth are associated with a higher risk of overweight and obesity among young adults, the consequences of which can significantly affect the length and quality of life in adulthood and old age [5,7,10,11,12,13,14,15]. The diet of the student population is characterized by an insufficient intake of raw fruits and vegetables, milk and dairy products, fish, and nuts, while protein intake is generally higher than the recommended level. Students most often consume proteins of animal origin, mainly from red meat and meat products [9]. As sources of carbohydrates, the student population most often chooses pasta, white bread, breakfast cereals, pastries, biscuits, and white rice, which provide them with the necessary energy to fulfill their study obligations. The proportion of foods made from whole grains in the diet of students is insufficient [16].

Research shows that the prevalence of fast-food consumption in the student population is high. “Fast food”, which includes baked or fried meat, is most often consumed by students with fried potatoes, along with the frequent consumption of meat products [17]. “Fast food” is rich in saturated fats, trans-fats, and has a high salt content, which can be a risk for the development of NCDs. The share of unsaturated fats in the diet of students is low [18,19].

Many factors influence the way students eat. Research has shown that students most often choose meals based on price, availability, and ease of preparation [20]. Habits acquired in the early period of life under the influence of family eating habits also represent one of the most significant factors affecting students’ nutrition [11]. Knowledge about nutrition, dietary guidelines, food groups and the nutrients they contain, the selection and adequate preparation of food, and knowledge about the health consequences of unhealthy nutrition significantly influence students’ eating habits [21].

Students who have a greater understanding of recommendations for proper nutrition do not always manage to apply them, due to various social influences, personal motivation, and already-established bad eating habits [6]. Study obligations and prolonged stress due to increased demands negatively influence students’ eating habits. Research shows that a higher level of nutrition knowledge (usually acquired through education at the faculties of health studies) is significantly associated with healthier nutritional habits [22,23,24].

Enhancing nutrition knowledge is posited as a key strategy to improve dietary habits, thereby fostering better health outcomes and reducing the risk of diet-related diseases. Since poor dietary habits are a leading risk factor for many chronic conditions, improving nutrition knowledge within the population is crucial for public health. By fostering healthier dietary behaviors and enhancing nutrition knowledge, we can help in reducing the incidence of diet-related diseases, thereby alleviating healthcare burdens and supporting long-term health and wellness outcomes [25].

To date, no research has been conducted on students’ nutrition knowledge at the university level in Serbia.

The aim of this study was to determine the nutrition knowledge of students from University of Novi Sad (Vojvodina, Serbia) in relation to gender, year of study, academic field of study, and nutritional status.

## 2. Materials and Methods

### 2.1. Study Design and Population

The research was conducted in the period from December 2021 to July 2022 as an observational, cross-sectional study among 898 students from the following 8 faculties of the University of Novi Sad: Faculty of Medicine; Faculty of Science; Faculty of Sports, Faculty of Philosophy; Academy of Arts; Faculty of Technology; Faculty of Agriculture; and Faculty of Technical Sciences. A convenience sampling method was used. The sample size for students from each individual faculty that provided a confidence interval of 95% and a margin of error of 5–8% was determined using the power analysis method [26].

### 2.2. Study Questionnaire

An online survey questionnaire generated using an online platform (Google Forms) that can be accessed from any device with an internet connection (PC or smartphone) was used as a research instrument. The survey was sent to students via social networks (Facebook and Instagram). Consent for participation in the study was confirmed by filling out the survey and submitting the answers. The survey was voluntary and anonymous, and respondents could withdraw from participation in the research at any time. Only fully completed questionnaires were recorded in the database for further analysis. Upon the study’s completion, the database was downloaded as a Microsoft Excel spreadsheet. Google’s privacy policy ensured the anonymity of the respondents.

### 2.3. Variables

The survey consisted of three parts.

In the first part of the survey, data on the general characteristics of the respondents were collected, including their gender, year of study, and faculty attended, while body mass index (BMI) was calculated based on the self-reported data on body height and body weight. According to the BMI value, the subjects were classified according to the following WHO recommendations: <18.5 kg/m^2^ (underweight); 18.5–24.99 kg/m^2^ (normal weight); 25–29.99 kg/m^2^ (overweight); and ≥30 kg/m^2^ (obesity).

In the second part of the survey, respondents rated their adherence to a proper diet (1—I do not adhere at all, 5—I adhere entirely).

Self-assessment of knowledge on a Likert scale of 1 to 5 was performed for each of the following domains: dietary recommendations; food groups and the nutrients they contain; healthy food choices; and diet, disease and weight associations.

In the third part of the survey, the students’ knowledge of healthy nutrition was assessed (determined) using the General Nutrition Knowledge Questionnaire (GNKQ) [27]. Previously, the questionnaire had demonstrated satisfactory reliability and validity among the student population [28]. The questionnaire showed good internal consistency (Cronbach’s alpha = 0.878).

The results were scored by giving each correct answer 1 point and each incorrect answer 0 points (Supplement File S1). Total and individual knowledge in the following 4 domains were examined: dietary recommendations (18 questions); food groups and the nutrients they contain (35 questions); healthy food choice (13 questions); diet, disease, and weight associations (16 questions).

The knowledge score from each domain is shown as a percentage of correct answers, and the total (determined) knowledge for healthy nutrition score is shown as a percentage of all correct answers included in the questionnaire.

### 2.4. Statistical Analysis

Qualitative (nominal) variables were described by absolute numbers (n) and frequency distribution (%), and quantitative (numerical) variables were described using arithmetic mean (X) and standard deviation (SD).

The chi-square test (χ^2^ test) was used to compare qualitative (nominal) data frequencies. For tables larger than 2 × 2, a Cramer’s V measured the association strength, while the Phi coefficient was employed for 2 × 2 tables.

Skewness and kurtosis values of numerical variables ranged from −2 to +2 and were considered adequate indicators of normal univariate distribution [29]. Graphical analysis of the distribution of the numerical variables, using histograms and quantile–quantile (Q–Q) charts, confirmed the assumption of normality of the distribution. A satisfactory equality of variance between the groups investigated was determined by analysis of the scatter diagram. The sample was large enough, and the results for the examined variables between the groups were mutually independent.

By satisfying the above assumptions, parametric tests were used. A T-test of independent samples was used to compare the mean values of numerical variables between two groups, and a one-way analysis of variance (one-way ANOVA) was used to compare the mean values between three or more groups.

Pearson’s correlation was used to examine the relationship between two numerical variables. As a measure of the size of the correlation, the interpretation, according to Cohen, was used, as 0.10–0.29 small, 0.30–0.49 medium and 0.5–1 large.

To examine the relationship between the dependent variables (the high level of total knowledge, high level of knowledge of dietary recommendations, high level of knowledge of food groups and the nutrients they contain, high level of knowledge of healthy food choices, and high level of knowledge of diet, disease and weight associations) and the independent variables (gender, year of study, attended faculty, and nutritional status), binary logistic regression (BLR) was used.

Univariate BLR examined the individual influence of each independent variable on the outcome variable, with the reference category being “≥75 score”. Multivariate BLR estimated the probability of a binary outcome based on one or more predictor variables, allowing for the examination of the combined effect of multiple independent variables on the likelihood of a high level of nutrition knowledge as the dependent variable. The results are presented as an odds ratio (OR) with a 95% confidence interval (CI), and an associated probability level p.

SPSS Statistics for Windows ver. 24 (IBM Corporation, Armonk, NY, USA) was used for statistical data analysis. Differences for which the *p*-value was less than 0.05 were considered statistically significant.

### 2.5. Ethical Aspects of the Research

The study was conducted according to the guidelines of the Declaration of Helsinki. The Ethics Committee of the Faculty of Medicine University of Novi Sad waived the need for ethical approval for the study since the research did not incorporate invasive methods and did not violate the privacy of the respondents. Informed consent was obtained from all subjects.

## 3. Results

The sample included 898 participants, composed of 655 (72.9%) female students and 243 (27.1%) male students. The average age of the participants was 22.87 ± 3.092 (for male students 23.02 ± 2.845, and for female students 22.81 ± 3.179), with no statistically significant difference in the age of participants in relation to gender (t = −0.897; *p* = 0.37). There was no statistically significant difference in the distribution of participants based on gender in relation to year of study (χ^2^ = 0.248; *p* = 0.619; fi = 0.017), nor in the distribution of participants based on year of study in relation to attended faculty (χ^2^ = 7.717; *p* = 0.353; fi = 0.093). The sample structure is presented in Table 1.

Self-assessed adherence rating to proper nutrition differed between students from different faculties (F = 2.883; *p* = 0.006). Students from the Faculty of Sport had the highest self-assessed rating of adherence to proper nutrition (3.73 ± 0.918), while students from the Faculty of Philosophy had the lowest (3.21 ± 0.958). Normal-weight students rated their adherence to proper nutrition at the highest degree (3.42 ± 0.867), whereas obese students rated at the lowest (2.54 ± 0.905) in relation to the nutritional status of other students (F = 10.247; *p* < 0.001). There was no statistically significant difference in the self-assessed rating of adherence to proper nutrition between female and male students (t = −0.3; *p* = 0.764) and between students from different years of study (t = −0.594; *p* = 0.553).

Considering the whole sample, students had the highest self-assessed knowledge of diet, disease, and weight associations (4.33 ± 0.862), followed by knowledge of healthy food choices (3.75 ± 1.041) and knowledge of food groups and the nutrients they contain (3.75 ± 1.035), while self-assessed knowledge of dietary recommendations was the lowest (3.35 ± 0.880) (not shown in tables).

A significant difference in self-assessed knowledge of dietary recommendations was found among students from different faculties (F = 2.342; *p* = 0.023). Students from the Faculty of Technology had the highest level of self-assessed knowledge of dietary recommendations (3.98 ± 0.739), while students from the Academy of Arts had the lowest (3.63 ± 1.070). There was no statistically significant difference in self-assessed knowledge of dietary recommendations in relation to gender (t = −1.281; *p* = 0.2), year of study (t = 0.812; *p* = 0.417), and nutritional status (F = 1.19; *p* = 0.312).

Male students had a significantly higher level of self-assessed knowledge about food groups and the nutrients they contain (3.86 ± 1.000) compared to female students (3.65 ± 1.067) (t = −2.551; *p* = 0.011). Self-assessed knowledge about food groups and the nutrients they contain differed between faculties (F = 3.879; *p* < 0.001). Students from the Faculty of Technology had the highest level of self-assessed knowledge about food groups and the nutrients they contain (3.94 ± 0.998), while students from the Faculty of Philosophy had the lowest (3.38 ± 1.220). No statistically significant difference was found in self-assessed knowledge about food groups and the nutrients they contain between students in relation to year of study (t = 0.232; *p* = 0.817) or students’ nutritional status (F = 2.496; *p* = 0.059).

Female students (3.66 ± 1.055) had a significantly lower level of self-assessed knowledge about healthy food choices compared to male students (3.84 ± 1.026) (t = −2.248; *p* = 0.025). Self-assessed knowledge about making healthy food choices differed between students with different nutritional status (F = 3.251; *p* = 0.021). Normal weight students had the highest level of self-assessed knowledge about healthy food choices (3.77 ± 1.016), while obese students had the lowest (3.27 ± 1.313). No statistically significant difference was found in self-assessed knowledge about healthy food choices between students in relation to year of study (t = 0.263; *p* = 0.793) and attended faculty (F = 1.405; *p* = 0.2).

Self-assessed knowledge about diet, disease, and weight associations significantly differed between faculties (F = 2.315; *p* = 0.024). Students from the Faculty of Medicine had the highest level of self-assessed knowledge (4.49 ± 0.689), while students from the Faculty of Sport had the lowest level of self-assessed knowledge about diet, disease and weight associations (4.13 ± 0.929). Self-assessed knowledge of diet, disease and weight associations significantly differed in relation to students’ nutritional status (F = 7.785; *p* < 0.001). Normal-weight students had the highest (4.37 ± 0.807), while obese students had the lowest self-assessed knowledge about diet, disease and weight associations (3.58 ± 1.270). No statistically significant difference was found in self-assessed knowledge about diet, disease, and weight associations in relation to gender (t = −0.086; *p* = 0.932) and year of study (t = 0.618; *p* = 0.537) (Table 2).

Considering the whole sample, students had a mean determined total knowledge about healthy nutrition of 63.51 ± 12.487, with the highest rating being for knowledge of healthy food choices (72.39 ± 16.935), followed by knowledge of diet, disease and weight associations (66.22 ± 15.759) and knowledge of dietary recommendations (62.57 ± 13.908), while knowledge about food groups and the nutrients they contain was the lowest among domains (59.07 ± 15.478) (not shown in tables).

The total determined knowledge about healthy nutrition correlated the most with the knowledge of food groups and the nutrients they contain (r = 0.88), followed by knowledge of diet, disease and weight associations (r = 0.793) and knowledge of dietary recommendations (r = 0.692), while it correlated the least with the knowledge of healthy food choices (r = 0.627). All correlations were high and statistically significant at the *p* < 0.001 level (not shown in tables).

In the fourth- to sixth-years of study, students had a significantly higher level of total knowledge about healthy nutrition (65.09 ± 12.151) compared to first- to third-year students (61.91 ± 12.631) (t = −3.849; *p* < 0.001). The difference in total knowledge by attended faculty was statistically significant (F = 12.798; *p* < 0.001). Students from the Faculty of Medicine had the highest total knowledge (68.35 ± 9.113), while students from the Academy of Arts had the lowest level of total knowledge about healthy nutrition (58.46 ± 12.419). No statistically significant difference was found in the total level of knowledge about healthy nutrition in relation to gender (t = 1.668; *p* = 0.096) or nutritional statuses (F = 0.287; *p* = 0.835).

Female students had a significantly higher level of knowledge about dietary recommendations (63.16 ± 13.560) compared to male students (60.97 ± 14.716) (t = 2.092; *p* = 0.037), and fourth- to sixth-year students had a significantly higher level of knowledge about dietary recommendations (63.78 ± 14.177) compared to first- to third-year students (61.34 ± 13.535) (t = −2.64; *p* = 0.008). The difference in knowledge about dietary recommendations in relation to attended faculty was statistically significant (F = 6.893; *p* < 0.001). The highest level of knowledge about dietary recommendations was among students from the Faculty of Medicine (66.87 ± 12.033), and the lowest was among students from the Faculty of Technical Sciences (58.54 ± 13.253). No statistically significant difference was found in the level of knowledge about dietary recommendations among students in relation to nutritional status (F = 2.156; *p* = 0.092).

Students in their fourth- to sixth-year of study had a significantly higher level of knowledge about food groups and the nutrients they contain (60.93 ± 15.425) compared to first- third-year students (57.18 ± 15.320) (t = −3.653; *p* < 0.001). The highest level of knowledge about food groups and the nutrients they contain (65.03 ± 15.698) was found among students from the Faculty of Technology, and the lowest was among students from the Faculty of Philosophy (52.63 ± 16.995), with the significant difference in relation to attended faculties (F = 9.433; *p* < 0.001). No statistically significant difference was found in the level of knowledge about food groups and the nutrients they contain in relation to gender (t = −0.766; *p* = 0.444) or nutritional statuses (F = 0.376; *p* = 0.774).

Female students had a statistically significantly higher level of knowledge about healthy food choices (73.75 ± 15.429) compared to male students (68.72 ± 20.031) (t = 3.542; *p* < 0.001). The highest level of knowledge of healthy food choices (76.45 ± 15.227) was among students from the Faculty of Science, and the lowest was among students from the Academy of Arts (63.51 ± 20.429), with the significant difference in relation to attended faculties (F = 7.939; *p* < 0.001). No statistically significant difference was found in the level of knowledge about healthy food choices in relation to year of study (t = −0.857; *p* = 0.392) or nutritional status (F = 0.371; *p* = 0.774).

Female students had a significantly higher level of knowledge about diet, disease, and weight associations (67.15 ± 15.085) compared to male students (63.73 ± 17.233) (t = 2.729; *p* = 0.037), and fourth- to sixth-year students had a significantly higher level of knowledge (68.33 ± 14.598) to first- to third-year students (64.08 ± 16.598) (t = −4.074; *p* < 0.001). Students from the Faculty of Medicine had the highest level of knowledge (73.02 ± 11.623), while students from the Faculty of Philosophy had the lowest level of knowledge about diet, disease and weight associations (60.78 ± 15.742), with significant differences found in relation to attended faculties (F = 13.404; *p* < 0.001). No statistically significant difference was found in the level of knowledge of diet, disease, and weight associations in relation to students’ nutritional status (F = 0.729; *p* = 0.535) (Table 3).

The total determined knowledge about healthy nutrition correlated the most with self-assessed knowledge of food groups and the nutrients they contain (r = 0.403), followed by knowledge of diet, disease, and weight associations (r = 0.269), and knowledge of healthy food choices (r = 0.267), while it correlated least with self-assessed knowledge of dietary recommendations (r = 0.22). All correlations were statistically significant at the *p* < 0.001 level (not shown in tables).

The total determined knowledge about healthy nutrition correlated the most with self-assessed knowledge of food groups and the nutrients they contain when analyzed individually for gender, academic year, attended faculty (except for students at the Academy of Arts, Faculty of Technology, and Faculty of Medicine), and nutritional status (except for underweight and obese students). The correlation between the total determined knowledge about healthy nutrition and self-assessed knowledge of healthy food choices was highest among students from the Faculty of Medicine and students at the Academy of Arts, as well as among obese and underweight students. However, for students at the Faculty of Technology, the highest correlation was observed between knowledge of diet, disease and weight associations, and the total determined knowledge about healthy nutrition (Table 4).

When considering the entire sample, 15.1% of students had a high level of total knowledge about healthy nutrition, 20.9% had a high level of knowledge of dietary recommendations, 12.2% had a high level of knowledge of food groups and the nutrients they contain, 57.6% had a high level of knowledge of healthy food choices, and 33.5% had a high level of knowledge of diet, disease and weight associations (not shown in tables).

Students in their fourth- to sixth-year of study more frequently had a high level of total knowledge about healthy nutrition (19.7%) compared to first- to third-year students (10.5%) (χ^2^ = 14.632; *p* < 0.001; fi = 0.128). A statistically significant difference was found in the frequency of high-level total knowledge of healthy nutrition between students from different faculties (χ^2^ = 33.847; *p* < 0.001; fi = 0.194). Students from the Faculty of Technology had the highest percentage of high-level total knowledge of healthy nutrition (31.5%), while students from the Faculty of Agriculture had the lowest (6.3%). No statistically significant difference was found in the frequency of high-level total knowledge of healthy nutrition in relation to gender (χ^2^ = 0.028; *p* = 0.867; fi = 0.006) and nutritional status (χ^2^ = 1.851; *p* = 0.604; fi = 0.045).

Students in their fourth- to sixth-year of study more frequently had a high level of knowledge of dietary recommendations (24.3%) compared to first- to third-year students (17.5%) (χ^2^ = 6.359; *p* = 0.012; fi = 0.084). A statistically significant difference was found in the frequency of high-level knowledge of dietary recommendations between students from different faculties (χ^2^ = 25.721; *p* = 0.001; fi = 0.169). Students from the Faculty of Technology had the highest percentage of a high level of knowledge of dietary recommendations (31.5%), while students from the Academy of Arts had the lowest (9.3%). No statistically significant difference was found in the frequency of a high level of knowledge of dietary recommendations in relation to gender (χ^2^ = 2.113; *p* = 0.146; fi = 0.049) and nutritional status (χ^2^ = 1.522; *p* = 0.677; fi = 0.041).

Students in their fourth- to sixth-year of study more frequently had a high level of knowledge of food groups and the nutrients they contain (14.8%) compared to first- to third-year students (9.6%) (χ^2^ = 5.608; *p* = 0.018; fi = 0.079). A statistically significant difference was found in the frequency of high-level knowledge of food groups and the nutrients they contain between students from different faculties (χ^2^ = 18.110; *p* = 0.011; fi = 0.142). Students from the Faculty of Technology had the highest percentage of high-level knowledge of food groups and the nutrients they contain (25.9%), while students from the Faculty of Philosophy had the lowest (6.4%). No statistically significant difference was found in the frequency of a high level of food groups and the nutrients they contain in relation to gender (χ^2^ = 2.04; *p* = 0.153; fi = 0.048) and nutritional status (χ^2^ = 1.456; *p* = 0.693; fi = 0.04).

Female students had a high level of knowledge of healthy food choices (60.5%) more frequently compared to male students (49.8%) (χ^2^ = 8.251; *p* = 0.004; fi = 0.096). A statistically significant difference was found in the frequency of high-level knowledge of healthy food choices between students from different faculties (χ^2^ = 35.732; *p* < 0.001; fi = 0.199). Students from the Faculty of Medicine had the highest percentage of high-level knowledge of healthy food choices (67.8%), while students from the Faculty of Sport had the lowest percentage (36.7%). No statistically significant difference was found in the frequency of a high level of knowledge of healthy food choices in relation to year of study (χ^2^ = 0.09; *p* = 0.764; fi = 0.01) and nutritional status (χ^2^ = 1.197; *p* = 0.754; fi = 0.037).

Female students more frequently had a high level of knowledge of diet, disease, and weight associations (35.7%) compared to male students (27.6%) (χ^2^ = 5.287; *p* = 0.021; fi = 0.077). Students in their fourth- to sixth-year of study were more likely to have a high level of knowledge of diet, disease, and weight associations (39.8%) compared to first- to third-year students (27.1%) (χ^2^ = 16.231; *p* < 0.001; fi = 0.134). A statistically significant difference was found in the frequency of high-level knowledge of diet, disease and weight associations between students from different faculties (χ^2^ = 86.251; *p* < 0.001; fi = 0.31). Students from the Faculty of Medicine had the highest percentage of a high level of knowledge of diet, disease and weight associations (55.0%), while students from the Academy of Arts had the lowest (16.4%). No statistically significant difference was found in the frequency of a high level of knowledge of diet, disease and weight associations in relation to nutritional status (χ^2^ = 7.462; *p* = 0.059; fi = 0.091) (Table 5).

The results of univariate BLR analysis showed that year of study and attended faculty were significant predictors of having a high level of total knowledge about healthy nutrition. The odds of having a high level of total knowledge about healthy nutrition were 2.081 times higher for fourth- to sixth-year students (95% CI:1.422–3.046; *p* < 0.001), as well as 2.361 times higher for students from the Faculty of Medicine (95% CI: 1.273–4.378; *p* = 0.006), 2.289 times higher for students from the Faculty of Science (95% CI: 1.154–4.541; *p* = 0.018), and 4.166 times higher for students from the Faculty of Technology (95% CI: 1.903–9.121; *p* < 0.001).

In the multivariate BLR model, the year of study and attended faculty remained as predictors of having a high level of total knowledge about healthy nutrition.

The odds of having a high level of total knowledge about healthy nutrition were 2.025 times higher for fourth- to sixth-year students (95% CI:1.370–2.993; *p* < 0.001), as well as 2.431 times higher for students from the Faculty of Medicine (95% CI: 1.271–4.649; *p* = 0.007), 2.347 times higher for students from the Faculty of Science (95% CI: 1.156–4.767; *p* = 0.018), and 4.507 times higher for students from the Faculty of Technology (95% CI: 1.987–10.223; *p* < 0.001).

Gender and nutritional status were not significant predictors of a high level of total knowledge about healthy nutrition (Table 6).

The results of univariate BLR analysis showed that year of study and attended faculty were significant predictors of having a high level of knowledge of dietary recommendations. The odds of having a high level of knowledge of dietary recommendations for fourth- to sixth-year students were 1.517 times higher (95% CI:1.096–2.101; *p* = 0.012), as well as 2.666 times higher for students from the Faculty of Medicine (95% CI: 1.543–4.604; *p* < 0.001), 1.863 times higher for students from the Faculty of Philosophy (95% CI: 1.005–3.452; *p* = 0.048), and 3.009 times higher for students from the Faculty of Technology (95% CI: 1.432–6.323; *p* = 0.004).

In the multivariate BLR model, year of study and attended faculty remained as predictors of having high level of knowledge of dietary recommendations.

The odds of having a high level of knowledge of dietary recommendations were 1.489 times higher for fourth- to sixth-year students than for first- to third-year students (95% CI:1.068–2.074; *p* = 0.019), as well as 2.497 times higher for students from the Faculty of Medicine (95% CI: 1.419–4.394; *p* = 0.002) and 2.820 times higher for students from the Faculty of Technology (95% CI: 1.317–6.042; *p* = 0.008). Attending the Faculty of Philosophy was not a significant predictor of a high level of knowledge of dietary recommendations in this model.

Gender and nutritional status were not significant predictors of a high level of knowledge of dietary recommendations (Table 7).

The results of univariate BLR analysis showed that year of study and attended faculty were significant predictors of having a high level of knowledge of food groups and the nutrients they contain. The odds of having a high level of knowledge of food groups and the nutrients they contain were 1.631 times higher for fourth- to sixth-year students (95% CI:1.085–2.452; *p* = 0.019), as well as 2.953 times higher for students from the Faculty of Technology (95% CI: 1.328–6.568; *p* = 0.008).

In the multivariate BLR model, year of study and attended faculty remained and gender was added as a significant predictor of having a high level of knowledge of food groups and the nutrients they contain.

The odds of having a high level of knowledge of food groups and the nutrients they contain was 1.730 times higher for male students than for female students (95% CI:1.075–2.785; *p* = 0.024), and were 1.580 times higher for fourth- to sixth-year students than for first- to third-year students (95% CI:1.042–2.395; *p* = 0.031), as well as 3.622 times higher for students from the Faculty of Technology (95% CI: 1.568–8.370; *p* = 0.003).

Nutritional status was not a significant predictor of a high level of knowledge of food groups and the nutrients they contain (Table 8).

The results of univariate BLR analysis showed that gender and attended faculty were significant predictors of having a high level of knowledge of healthy food choices. The odds of having a high level of knowledge of healthy food choices were 2.021 times higher for students from the Faculty of Medicine (95% CI: 1.331–3.068; *p* = 0.001), and 1.922 times higher for students from the Faculty of Science (95% CI:1.183–3.124; *p* = 0.008). Male gender was a significant negative predictor of a high level of knowledge of healthy food choices (OR: 0.649; 95% CI:0.482–0.872; *p* = 0.004).

In the multivariate BLR model, only attended faculty remained as a predictor of having a high level of knowledge of healthy food choices.

The odds of having a high level of knowledge of healthy food choices were 1.896 times higher for students from the Faculty of Medicine (95% CI: 1.229–2.925; *p* = 0.004) and 1.826 times higher for students from the Faculty of Science (95% CI: 1.112–2.996; *p* = 0.017). Gender was not a significant predictor of a high level of knowledge of healthy food choices in this model.

Year of study and nutritional status were not significant predictors of a high level of knowledge of healthy food choices (Table 9).

The results of univariate BLR analysis showed that gender, year of study and attended faculty were significant predictors of having a high level of knowledge of diet, disease and weight associations. The odds of having a high level of knowledge of diet, disease, and weight associations were 1.777 times higher for fourth- to sixth-year students than for first- to third-year students (95% CI:1.342–2.354; *p* < 0.001), 3.898 times higher for students from the Faculty of Medicine (95% CI: 2.481–6.125; *p* < 0.001), and 2.556 times higher for students from the Faculty of Technology (95% CI:1.328–4.917; *p* = 0.005). Male gender was a significant negative predictor of a high level of knowledge of diet, disease, and weight associations (OR: 0.685; 95% CI:0.496–0.947; *p* = 0.022).

In the multivariate BLR model, year of study and attended faculty remained as predictors, and nutritional status was added as a significant predictor of a high level of knowledge of diet, disease, and weight associations.

The odds of having a high level of knowledge of diet, disease, and weight associations were 1.771 times higher for fourth- to sixth-year students than for first- to third-year students (95% CI:1.314–2.388; *p* < 0.001), 3.628 times higher for students from the Faculty of Medicine (95% CI: 2.261–5.820; *p* < 0.001) and 2.340 times higher for students from the Faculty of Technology (95% CI: 1.189–4.606; *p* = 0.014). Being overweight was a significant negative predictor of a high level of knowledge of diet, disease, and weight associations (OR: 0.436; 95% CI:0.223–0.852; *p* = 0.015). Gender was not a significant predictor of a high level of knowledge of diet, disease, and weight associations in this model (Table 10).

## 4. Discussion

To our knowledge, this is the first study to examine general nutrition knowledge among university students in Serbia. A strong understanding and knowledge of nutrition can have a significant impact on people’s eating habits and overall wellbeing [30]. Mostafazadeh et al. [30] determined that a 44% variance in the eating behavior of nursing students from Iran was explained by nutritional literacy, and that higher scores of nutrition knowledge correlated with better nutritional habits. Factors such as education levels, gender, cultural background, socioeconomic status, and personal health goals can all influence how individuals perceive and approach nutrition [31,32,33]. Education about the importance of a balanced diet, the role of nutrients in the body, and how to make healthy food choices can empower them to make positive changes in their eating habits and lifestyle [34,35]. It can also contribute to reducing the risk of nutrition-related health issues and improving the overall quality of life.

Our study shows that attended faculty (academic field of study) is an important variable associated with knowledge of nutrition, whether for the self-assessed measure score or the determined score of nutrition knowledge. Also, we confirmed that a high level of knowledge was significantly associated with the attended faculty. Students from the Faculty of Medicine, Faculty of Technology, and Faculty of Sport rated themselves as having a high level of nutrition knowledge, which was consistent with the results of the determined score. Several possible reasons may explain this. The first of these is curriculum emphasis. These disciplines may include nutrition education as part of their curriculum, either as a core subject or as an elective course. Medical students, for example, often study nutrition as part of their training due to its importance in healthcare [22]. Students in the mentioned fields may recognize the importance of nutrition in their respective professions. For instance, medical students need to understand nutrition to provide comprehensive patient care, while sports students focus on optimizing physical performance through proper nutrition [22,36]. The second reason is practical application. The nature of their disciplines may require the practical application of nutrition knowledge. For instance, engineers working on food technology or biomedical devices need to understand nutritional principles for product development [37]. In addition, students within these disciplines may discuss and share information about nutrition (peer influence), leading to a collective degree of high knowledge within the group [38]. Although Ozdoğan and Ozcelik [39] found that university students in Turkey receiving sports education, who were expected to continue their professional lives in sport-related fields, were determined to have a lack of knowledge on nutrition, it is acknowledged that it is necessary to strengthen nutrition education.

Our data shows that gender is another significant variable in knowledge of nutrition for both the self-assessed and determined categories, which is mainly reflected in knowledge of food groups and the nutrients they contain and knowledge of healthy food choices. More interestingly, male students had higher self-assessed knowledge of healthy food choices but, in the determined results, female students had a better knowledge of healthy food choices, and this difference was highly significant. This aligns with existing research showing that gender can influence dietary preferences, nutritional needs, and attitudes toward food and health [40,41]. Similarly, Egele and Stark [42] conducted a cross-sectional study among 212 German participants and found most of the anticipated gender differences in food choice, and some differences in health beliefs. In a recent study in 2024 Rome (Italy) with 2198 participants, Feraco et al. [43] came to the similar conclusion that gender dynamics significantly influence food choice and eating habits; women tend to choose healthier foods and eat regular meals, while men show preferences for specific tastes. In addition, this gender difference is also widely observed in other university students, such as in those from Norway [44], China [45], the USA [18], and Croatia [46]. Female students generally demonstrate a higher level of interest in monitoring their dietary intake and are more inclined to receive nutrition education compared to their male counterparts [47].

Our study also revealed two interesting findings. There were no significant differences in self-assessed nutrition knowledge among students of different academic years, but significant differences were observed in their actual determined nutrition knowledge (higher academic years scored higher than lower academic years). Also, we confirmed that a high level of knowledge (≥75 scores) was significantly associated with the year of study. Obese students perceived themselves to have lower nutrition knowledge levels, but this difference was not significant in actual measurements (determined nutrition knowledge score). A supporting study had a similar result, which showed that a higher year of study was associated with higher functional nutrition literacy levels in university students from Norway [44]. Several studies reported similar findings [46,48,49,50]. Higher academic years typically involve more advanced coursework and greater exposure to nutrition-related topics [51]. This exposure and education may contribute to a deeper understanding and knowledge of nutrition among students in higher academic years that develops imperceptibly, which could explain our results. However, this is not certain, as higher education does not automatically equate to high nutritional or health literacy. A population study conducted among Danish people found that, despite being a relatively well-educated group, they have a high prevalence of insufficient health knowledge [52].

The findings of our study indicate varying levels of knowledge in relation to different domains among students. Overall, students demonstrated a moderate level of total knowledge about healthy nutrition, with the highest level being understanding of healthy food choices, followed by knowledge of diet, disease, and weight associations, and knowledge of dietary recommendations. However, the level of knowledge about food groups and the nutrients they contain was notably lower compared to other domains. This discrepancy suggests potential gaps in students’ understanding of fundamental nutritional concepts. Furthermore, the results of our study indicate that the self-assessed knowledge of food groups and nutrients they contain exhibited the strongest correlation with total determined knowledge about healthy nutrition, underscoring the importance of addressing this particular area of deficiency in nutrition education. Interestingly, while a substantial proportion of students demonstrated a high level of knowledge in certain domains, such as healthy food choices and diet–disease associations, fewer students exhibited a high level of knowledge in areas such as dietary recommendations and food groups. Potential explanations of the obtained results were made possible by the individual analysis of the association of specific independent variables (gender, year of study, faculty attended, BMI) with individual domains of nutrition knowledge. In future studies, will be necessary to examine the connection between nutrition knowledge and eating habits, as well as the potential mediating and moderating effects of independent variables (demographic, social-economic variables, personality traits, motivational factors, etc.,) in order to further explain the dynamic relationship between knowledge/awareness and nutritional (health) behavior.

The strength of conducted research is such that, for the first time, a study was conducted on nutrition knowledge among students of all faculties of the second-largest university in Serbia. Due to their similar cultural, demographic, and social-economic characteristics, the results of our study are complementary to studies from the countries of the Western Balkans and Southeast Europe, which makes our study the basis for future investigations into the effectiveness of various educational programs and public health interventions aimed at enhancing nutrition literacy and implementing preventive measures to improve the dietary habits of university students. The implications of our findings extend to the potential applications of our results in informing national or international public health initiatives, particularly those targeting future student populations. Some of the proposed educational and policy recommendations can include integrating mandatory nutrition literacy modules across various university programs to reach students in non-health fields and developing interdisciplinary workshops to enhance nutrition knowledge. These initiatives could help foster healthier dietary behaviors, reduce diet-related risks, and serve as a foundation for broader public health strategies within universities and similar settings in the region.

Our study has several limitations. Firstly, the cross-sectional nature of the study design limits our ability to establish causal relationships or assess changes in knowledge levels over time. Secondly, the reliance on self-reported data introduces potential biases, despite efforts to ensure anonymity and confidentiality. Finally, the study was only conducted in one university, so the generalization of our findings for all students in the country is not possible [53].

## 5. Conclusions

Significant differences in students’ determined knowledge levels were determined across various domains of healthy nutrition. The highest level of nutrition knowledge was in healthy food choices and the lowest was in understanding food groups and nutrient composition. The total determined knowledge about healthy nutrition correlated the most with self-assessed knowledge of food groups and least with knowledge of dietary recommendations. Female gender and higher years of study were associated with higher nutrition knowledge. Students from the Faculty of Medicine had the highest nutrition knowledge, followed by students from the Faculty of Technology and the Faculty of Science in relation to students from other faculties. Findings highlight the need for targeted interventions aimed at improving students’ understanding of specific nutritional concepts, ultimately empowering them to make informed dietary decisions for lifelong health and wellbeing.

## Figures and Tables

**Table 1:** Sample structure.

Variables		*n*	%
Gender	Female	655	72.9
	Male	243	27.1
Year of study	1–3	446	49.7
	4–6	452	50.3
Faculty	Faculty of Medicine	242	26.9
	Faculty of Science	129	14.4
	Faculty of Sport	60	6.7
	Faculty of Philosophy	140	15.6
	Academy of Arts	43	4.8
	Faculty of Technology	54	6.0
	Faculty of Agriculture	79	8.8
	Faculty of Technical Sciences	151	16.8
Nutritional status	Underweight	70	7.8
	Normal weight	658	73.3
	Overweight	144	16.0
	Obese	26	2.9

**Table 2:** The self-assessed measure of adherence to proper nutrition, knowledge of dietary recommendations, knowledge of food groups and the nutrients they contain, knowledge of healthy food choices, and knowledge of diet, disease and weight associations of students from the University of Novi Sad (Serbia) (*n* = 898) in relation to gender, year of study, attended faculty and nutritional status.

Self-Assessed		Adherence to Proper Nutrition			Knowledge of Dietary Recommendations			Knowledge of Food Groups and the Nutrients They Contain			Knowledge of Healthy Food Choices			Knowledge of Diet, Disease and Weight Associations		
Variables		X	SD	*p* *	X	SD	*p* *	X	SD	*p* *	X	SD	*p* *	X	SD	*p* *
Gender	Female	3.34	0.896	0.764	3.85	0.920	0.2	3.65	1.067	**0.011**	3.66	1.055	**0.025**	4.32	0.847	0.932
	Male	3.36	0.891		3.94	0.896		3.86	1.000		3.84	1.026		4.33	0.904	
Year of study	1–3	3.33	0.888	0.553	3.90	0.927	0.471	3.71	1.029	0.817	3.72	1.041	0.793	4.34	0.797	0.537
	4–6	3.37	0.902		3.85	0.901		3.71	1.076		3.70	1.060		4.31	0.923	
Faculty	Faculty of Medicine	3.34	0.845	**0.006**	3.96	0.846	**0.023**	3.91	0.905	**<0.001**	3.84	0.908	0.2	4.49	0.689	**0.024**
	Faculty of Science	3.47	0.820		3.90	0.818		3.71	0.947		3.75	1.046		4.29	0.744	
	Faculty of Sport	3.73	0.918		3.70	1.013		3.70	0.979		3.68	1.033		4.13	0.929	
	Faculty of Philosophy	3.21	0.958		3.69	0.968		3.38	1.220		3.53	1.062		4.25	0.866	
	Academy of Arts	3.40	1.003		3.63	1.070		3.63	1.113		3.60	1.237		4.14	1.037	
	Faculty of Technology	3.41	0.836		3.98	0.739		3.94	0.998		3.81	1.047		4.22	0.883	
	Faculty of Agriculture	3.28	0.973		3.90	1.139		3.68	1.236		3.72	1.229		4.30	1.159	
	Faculty of Technical Sciences	3.23	0.868		3.97	0.848		3.65	1.047		3.64	1.092		4.33	0.907	
Nutritional status	Underweight	3.33	0.880	**<0.001**	3.70	0.983	0.312	3.57	1.098	0.059	3.51	1.126	**0.021**	4.21	0.866	**<0.001**
	Normal weight	3.42	0.867		3.90	0.903		3.74	1.041		3.77	1.016		4.37	0.807	
	Overweight	3.19	0.938		3.89	0.940		3.74	1.051		3.63	1.089		4.30	0.954	
	Obese	2.54	0.905		3.73	0.827		3.23	1.142		3.27	1.313		3.58	1.270	

* *p*-value calculated by using *t*-test for independent samples. Significant at *p* < 0.05.

**Table 3:** Determined total knowledge of healthy nutrition, knowledge of dietary recommendations, knowledge of food groups and the nutrients they contain, knowledge of healthy food choices, and knowledge of diet, disease and weight associations of students from the University of Novi Sad (Serbia) (*n* = 898) in relation to gender, year of study, attended faculty and nutritional status.

Determined		Total Knowledge of Healthy Nutrition			Knowledge of Dietary Recommendations			Knowledge of Food Groups and the Nutrients They Contain			Knowledge of Healthy Food Choices			Knowledge of Diet, Disease and Weight Associations		
Variables		X	SD	p *	X	SD	p *	X	SD	p *	X	SD	p *	X	SD	p *
Gender	Female	63.96	11.940	0.096	63.16	13.560	**0.037**	58.83	15.021	0.444	73.75	15.429	**<0.001**	67.15	15.085	**0.037**
	Male	62.29	13.807		60.97	14.716		59.72	16.664		68.72	20.031		63.73	17.233	
Year of study	1–3	61.91	12.631	**<0.001**	61.34	13.535	**0.008**	57.18	15.320	**<0.001**	71.90	17.410	0.392	64.08	16.598	**<0.001**
	4–6	65.09	12.151		63.78	14.177		60.93	15.425		72.87	16.459		68.33	14.598	
Faculty	Faculty of Medicine	68.35	9.113	**<0.001**	66.87	12.033	**<0.001**	63.40	11.694	**<0.001**	76.16	13.694	**<0.001**	73.02	11.623	**<0.001**
	Faculty of Science	64.53	11.423		60.77	13.479		60.40	14.806		76.45	15.227		67.26	12.627	
	Faculty of Sport	60.10	15.768		62.69	16.400		56.48	17.662		65.00	21.545		60.87	22.457	
	Faculty of Philosophy	59.08	13.087		61.75	14.138		52.63	16.995		70.00	18.159		60.78	15.742	
	Academy of Arts	58.46	12.419		59.56	11.837		53.82	15.735		63.51	20.429		62.13	14.283	
	Faculty of Technology	68.20	12.359		66.26	14.229		65.03	15.698		76.07	12.562		70.28	15.366	
	Faculty of Agriculture	62.24	12.705		60.48	15.939		59.93	14.505		68.55	16.535		63.71	17.259	
	Faculty of Technical Sciences	60.77	12.835		58.54	13.253		56.90	16.456		71.27	17.670		62.63	16.337	
Nutritional status	Underweight	63.02	11.344	0.835	61.91	12.451	0.092	57.59	14.311	0.774	73.30	16.132	0.774	66.67	15.636	0.535
	Normal weight	63.74	12.424		63.23	13.755		59.02	15.313		72.57	16.311		66.58	15.857	
	Overweight	62.83	13.351		60.11	15.404		59.96	16.749		71.10	19.728		64.81	14.869	
	Obese	62.73	12.563		61.11	11.547		59.34	15.903		72.49	18.484		63.74	18.471	

* *p* value calculated by using *t*-test for independent samples. Significant at *p* < 0.05.

**Table 4:** Correlation of determined and self-assessed knowledge of healthy nutrition by domains, in relation to gender, academic year, attended faculty, and nutritional status of students from the University of Novi Sad (Serbia) (*n* = 898).

Determined/Self-Assessed Knowledge		Adherence to Proper Nutrition	Knowledge of Dietary Recommendations	Knowledge of Food Groups and the Nutrients They Contain	Knowledge of Healthy Food Choices	Knowledge of Diet, Disease and Weight Associations
Gender	Female	0.213 **	0.255 **	0.394 **	0.237 **	0.257 **
	Male	0.061	0.143 *	0.434 **	0.378 **	0.318 **
Year of study	1–3	0.198 **	0.233 **	0.412 **	0.244 **	0.287 **
	4–6	0.132 **	0.220 **	0.395 **	0.291 **	0.251 **
Faculty	Faculty of Medicine	0.053	0.124	0.225 **	0.235 **	0.231 **
	Faculty of Science	0.017	0.200 *	0.518 **	0.218 *	0.102
	Faculty of Sport	0.234	0.067	0.317 *	0.245	0.271 *
	Faculty of Philosophy	0.234 **	0.341 **	0.426 **	0.385 **	0.322 **
	Academy of Arts	0.299	0.351 *	0.347 *	0.394 **	0.273
	Faculty of Technology	0.367 **	0.417 **	0.346 *	0.275 *	0.461 **
	Faculty of Agriculture	0.105	0.149	0.502 **	0.138	0.228 *
	Faculty of Technical Sciences	0.304 **	0.248 **	0.417 **	0.270 **	0.276 **
Nutritional status	Underweight	0.394 **	0.218	0.408 **	0.485 **	0.349 **
	Normal weight	0.142 **	0.236 **	0.390 **	0.255 **	0.287 **
	Overweight	0.233 **	0.181 *	0.455 **	0.171 *	0.152
	Obese	−0.178	−0.041	0.458 *	0.558 **	0.212

* *p* < 0.05; ** *p* < 0.001.

**Table 5:** Frequency of a high level of total knowledge of healthy nutrition, knowledge of dietary recommendations, knowledge of food groups and the nutrients they contain, knowledge of healthy food choices, and knowledge of diet, disease and weight associations of students from the University of Novi Sad (Serbia) (*n* = 898) in relation to gender, year of study, attended faculty and nutritional status.

High Level of Knowledge		Total Knowledge of Healthy Nutrition		Knowledge of Dietary Recommendations		Knowledge of Food Groups and the Nutrients They Contain		Knowledge of Healthy Food Choices		Knowledge of Diet, Disease and Weight Associations	
Variables		%	p *	%	p *	%	p *	%	p *	%	p *
Gender	Female	15.3	0.867	22.1	0.146	11.3	0.153	60.5	**0.004**	35.7	**0.021**
	Male	14.8		17.7		14.8		49.8		27.6	
Year of study	1–3	10.5	**<0.001**	17.5	**0.012**	9.6	**0.018**	58.1	0.764	27.1	**<0.001**
	4–6	19.7		24.3		14.8		57.1		39.8	
Faculty	Faculty of Medicine	20.7	**<0.001**	28.9	**0.001**	13.6	**0.011**	67.8	**<0.001**	55.0	**<0.001**
	Faculty of Science	20.2		18.6		16.3		66.7		31.0	
	Faculty of Sport	11.7		20.0		8.3		36.7		25.0	
	Faculty of Philosophy	8.6		22.1		6.4		55.7		16.4	
	Academy of Arts	9.3		9.3		9.3		46.5		16.3	
	Faculty of Technology	31.5		31.5		25.9		63.0		44.4	
	Faculty of Agriculture	6.3		12.7		10.1		45.6		29.1	
	Faculty of Technical Sciences	9.9		13.2		10.6		51.0		23.8	
Nutritional status	Underweight	14.3	0.604	20.0	0.677	8.6	0.693	60.0	0.754	37.1	0.059
	Normal weight	15.8		21.4		12.8		56.5		35.4	
	Overweight	11.8		20.8		11.1		61.1		24.3	
	Obese	19.2		11.5		15.4		57.7		26.9	

* *p* value calculated by using χ^2^ test for categorical variables. Significant at *p* < 0.05.

**Table 6:** Association between independent variables and a high level of total knowledge about healthy nutrition for students from the University of Novi Sad (Serbia) (*n* = 898).

High Level of Total Knowledge		Univariate Logistic Regression				Multivariate Logistic Regression			
Variables		OR	95% CI for OR		*p*	OR	95% CI for OR		*p*
Gender	Male	0.965	0.639	1.459	0.867	1.380	0.867	2.197	0.175
	Female	1.00				1.00			
Year of study	4–6	2.081	1.422	3.046	**<0.001**	2.025	1.370	2.993	**<0.001**
	1–3	1.00				1.00			
Faculty	Faculty of Medicine	2.361	1.273	4.378	**0.006**	2.431	1.271	4.649	**0.007**
	Faculty of Science	2.289	1.154	4.541	**0.018**	2.347	1.156	4.767	**0.018**
	Faculty of Sport	1.197	0.462	3.101	0.711	1.217	0.466	3.181	0.688
	Faculty of Philosophy	0.850	0.383	1.885	0.689	0.891	0.394	2.015	0.782
	Academy of Arts	0.930	0.292	2.963	0.902	0.897	0.278	2.894	0.855
	Faculty of Technology	4.166	1.903	9.121	**<0.001**	4.507	1.987	10.223	**<0.001**
	Faculty of Agriculture	0.613	0.214	1.752	0.361	0.590	0.204	1.705	0.330
	Faculty of Technical Sciences	1.00				1.00			
Nutritional status	Normal weight	1.126	0.559	2.272	0.740	1.033	0.499	2.141	0.930
	Overweight	0.803	0.347	1.859	0.609	0.713	0.294	1.725	0.452
	Obese	1.429	0.438	4.663	0.555	1.204	0.346	4.184	0.770
	Underweight	1.00				1.00			

Abbreviations: OR—odds ratio; CI—confidence interval.

**Table 7:** Association between independent variables and a high level of knowledge of dietary recommendations for students from the University of Novi Sad (Serbia) (*n* = 898).

High Level of Knowledge of Dietary Recommendations		Univariate Logistic Regression				Multivariate Logistic Regression			
Variables		OR	95% CI for OR		*p*	OR	95% CI for OR		*p*
Gender	Male	0.756	0.518	1.103	0.147	0.957	0.633	1.449	0.837
	Female	1.00				1.00			
Year of study	4–6	1.517	1.096	2.101	**0.012**	1.489	1.068	2.074	**0.019**
	1–3	1.00				1.00			
Faculty	Faculty of Medicine	2.666	1.543	4.604	**<0.001**	2.497	1.419	4.394	**0.002**
	Faculty of Science	1.497	0.784	2.858	0.221	1.426	0.737	2.758	0.292
	Faculty of Sport	1.637	0.744	3.602	0.220	1.615	0.731	3.566	0.236
	Faculty of Philosophy	1.863	1.005	3.452	**0.048**	1.785	0.951	3.347	0.071
	Academy of Arts	0.672	0.217	2.083	0.491	0.635	0.203	1.980	0.434
	Faculty of Technology	3.009	1.432	6.323	**0.004**	2.820	1.317	6.042	**0.008**
	Faculty of Agriculture	0.949	0.421	2.141	0.900	0.916	0.404	2.078	0.833
	Faculty of Technical Sciences	1.00				1.00			
Nutritional status	Normal weight	1.091	0.590	2.017	0.781	1.009	0.536	1.898	0.978
	Overweight	1.053	0.517	2.142	0.887	1.040	0.495	2.186	0.917
	Obese	0.522	0.137	1.989	0.341	0.520	0.132	2.051	0.350
	Underweight	1.00				1.00			

Abbreviations: OR—odds ratio; CI—confidence interval.

**Table 8:** The association between independent variables and a high level of knowledge of food groups and the nutrients they contain for students from the University of Novi Sad (Serbia) (*n* = 898).

High Level of Knowledge of Food Groups and the Nutrients They Contain		Univariate Logistic Regression				Multivariate Logistic Regression			
Variables		OR	95% CI for OR		*p*	OR	95% CI for OR		*p*
Gender	Male	1.365	0.889	2.096	0.154	1.730	1.075	2.785	**0.024**
	Female	1.00				1.00			
Year of study	4–6	1.631	1.085	2.452	**0.019**	1.580	1.042	2.395	**0.031**
	1–3	1.00				1.00			
Faculty	Faculty of Medicine	1.332	0.706	2.514	0.376	1.520	0.780	2.962	0.219
	Faculty of Science	1.641	0.816	3.297	0.164	1.872	0.910	3.852	0.089
	Faculty of Sport	0.767	0.268	2.196	0.621	0.770	0.267	2.218	0.628
	Faculty of Philosophy	0.580	0.247	1.358	0.209	0.666	0.280	1.587	0.359
	Academy of Arts	0.865	0.273	2.739	0.806	0.914	0.285	2.927	0.879
	Faculty of Technology	2.953	1.328	6.568	**0.008**	3.622	1.568	8.370	**0.003**
	Faculty of Agriculture	0.951	0.388	2.329	0.912	0.988	0.399	2.449	0.979
	Faculty of Technical Sciences	1.00				1.00			
Nutritional status	Normal weight	1.561	0.656	3.717	0.314	1.511	0.622	3.669	0.362
	Overweight	1.333	0.498	3.571	0.567	1.163	0.420	3.219	0.771
	Obese	1.939	0.500	7.516	0.338	1.515	0.374	6.137	0.561
	Underweight	1.00				1.00			

Abbreviations: OR—odds ratio; CI—confidence interval.

**Table 9:** Association between independent variables and a high level of knowledge of healthy food choices of students from the University of Novi Sad (Serbia) (*n* = 898).

High Level of Knowledge of Healthy Food Choices		Univariate Logistic Regression				Multivariate Logistic Regression			
Variables		OR	95% CI for OR		*p*	OR	95% CI for OR		*p*
Gender	Male	0.649	0.482	0.872	**0.004**	0.730	0.527	1.011	0.058
	Female	1.00				1.00			
Year of study	4–6	0.960	0.737	1.251	0.764	0.910	0.693	1.197	0.501
	1–3	1.00				1.00			
Faculty	Faculty of Medicine	2.021	1.331	3.068	**0.001**	1.896	1.229	2.925	**0.004**
	Faculty of Science	1.922	1.183	3.124	**0.008**	1.826	1.112	2.996	**0.017**
	Faculty of Sport	0.556	0.301	1.029	0.061	0.546	0.295	1.014	0.055
	Faculty of Philosophy	1.209	0.762	1.918	0.420	1.138	0.710	1.823	0.591
	Academy of Arts	0.836	0.424	1.648	0.604	0.815	0.410	1.619	0.558
	Faculty of Technology	1.634	0.863	3.092	0.131	1.484	0.773	2.850	0.236
	Faculty of Agriculture	0.805	0.466	1.389	0.435	0.760	0.437	1.321	0.331
	Faculty of Technical Sciences	1.00				1.00			
Nutritional status	Normal weight	0.867	0.525	1.433	0.578	0.894	0.533	1.499	0.670
	Overweight	1.048	0.584	1.878	0.876	1.300	0.704	2.398	0.402
	Obese	0.909	0.365	2.266	0.838	1.185	0.458	3.069	0.727
	Underweight	1.00				1.00			

Abbreviations: OR—odds ratio; CI—confidence interval.

**Table 10:** Association between independent variables and a high level of knowledge of diet, disease, and weight associations of students from the University of Novi Sad (Serbia) (*n* = 898).

High Level of Knowledge of Diet, Disease and Weight Associations		Univariate Logistic Regression				Multivariate Logistic Regression			
Variables		OR	95% CI for OR		*p*	OR	95% CI for OR		*p*
Gender	Male	0.685	0.496	0.947	**0.022**	0.985	0.683	1.419	0.934
	Female	1.00				1.00			
Year of study	4–6	1.777	1.342	2.354	**<0.001**	1.771	1.314	2.388	**<0.001**
	1–3	1.00				1.00			
Faculty	Faculty of Medicine	3.898	2.481	6.125	**<0.001**	3.628	2.261	5.820	**<0.001**
	Faculty of Science	1.436	0.846	2.436	0.180	1.303	0.755	2.248	0.341
	Faculty of Sport	1.065	0.532	2.131	0.859	1.088	0.539	2.197	0.814
	Faculty of Philosophy	0.628	0.351	1.125	0.118	0.570	0.313	1.039	0.066
	Academy of Arts	0.621	0.255	1.516	0.295	0.539	0.218	1.332	0.181
	Faculty of Technology	2.556	1.328	4.917	**0.005**	2.340	1.189	4.606	**0.014**
	Faculty of Agriculture	1.312	0.711	2.422	0.385	1.267	0.678	2.368	0.458
	Faculty of Technical Sciences	1.00				1.00			
Nutritional status	Normal weight	0.928	0.557	1.546	0.773	0.730	0.422	1.264	0.261
	Overweight	0.543	0.293	1.007	0.052	0.436	0.223	0.852	**0.015**
	Obese	0.623	0.231	1.683	0.351	0.460	0.159	1.331	0.152
	Underweight	1.00				1.00			

Abbreviations: OR—odds ratio; CI—confidence interval.

## Data Availability

The data presented in this study are available upon reasonable request from the corresponding author.

## Supplementary Materials

The following supporting information can be downloaded at: https://www.mdpi.com/article/10.3390/nu16223918/s1, File S1. General Nutrition Knowledge Questionnaire (GNKQ)—Scoring information.
