# Supplementary material for: Assessment of the General Nutrition Knowledge of Students from the University of Novi Sad (Vojvodina, Serbia)
Source: Nutrients. 2024 Nov 16;16(22):3918. doi: 10.3390/nu16223918 (PMC11597663; doi:10.3390/nu16223918)
Supplement: Supplementary file 1 [file nutrients-16-03918-s001.zip › nutrients-3319964-supplementary.pdf]

GENERAL NUTRITION KNOWLEDGE QUESTIONNAIRE

Section 1: Knowledge of dietary recommendations

1. Do health experts recommend that people should be eating more, the same amount, or less of the following foods? (tick one box per food)

	More	Same	Less	Not Sure
Fruit	✓	☐	☐	☐
Food and drinks with added sugar	☐	☐	✓	☐
Vegetables	✓	☐	☐	☐
Fatty foods	☐	☐	✓	☐
Processed red meat	☐	☐	✓	☐
Wholegrains	✓	☐	☐	☐
Salty foods	☐	☐	✓	☐
Water	✓	☐	☐	☐

2. How many servings of fruit and vegetables per day do experts advise people to eat as a minimum? (One serving could be, for example, an apple or a handful of chopped carrots)
(tick one)

2	☐
3	☐
4	☐
5 or more	✓
Not sure	☐

3. Which of these types of fats do experts recommend that people should eat less of?
(tick one box per food)

	Eat less	Not eat less	Not sure
Unsaturated fats	☐	✓	☐
Trans fats	✓	☐	☐
Saturated fats	✓	☐	☐

4. Which type of dairy foods do experts say people should drink? (tick one)

Full fat (e.g. full fat milk)	☐
Reduced fat (e.g. skimmed and semi-skimmed milk)	✓
Mixture of full fat and reduced fat	☐
Neither, dairy foods should be avoided	☐
Not sure	☐

5. How many times per week do experts recommend that people eat oily fish (e.g. salmon and mackerel)? (tick one)

- 1-2 times per week ✓
- 3-4 times per week ☐
- Every day ☐
- Not sure ☐

6. Approximately how many alcoholic drinks is the maximum recommended per day (The exact number depends on the size and strength of the drink)? (tick one)

- 1 drink each for men and women ✓
- 2 drinks each for men and women ☐
- 2 drinks for men and 1 drink for women ☐
- 3 drinks for men and 2 drinks for women ☐
- Not sure ☐

7. How many times per week do experts recommend that people eat breakfast? (tick one)

- 3 times per week ☐
- 4 times per week ☐
- Every day ✓
- Not sure ☐

8. If a person has two glasses of fruit juice in a day, how many of their daily fruit and vegetable servings would this count as? (tick one)

- None ☐
- One serving ✓
- Two servings ☐
- Three servings ☐
- Not sure ☐

9. According to the 'eatwell guide' (a guideline showing the proportions of food types people should eat to have a balanced and healthy diet), how much of a person's diet should be made up of starchy foods? (tick one)

- Quarter ☐
- Third ✓
- Half ☐
- Not sure ☐

Section 2: Knowledge of food groups and the nutrients they contain

1. Do you think these foods and drinks are typically high or low in added sugar? (tick one box per food)

	High in added sugar	Low in added sugar	Not sure
Diet cola drinks	☐	✓	☐
Natural yoghurt	☐	✓	☐
Ice cream	✓	☐	☐
Tomato ketchup	✓	☐	☐
Melon	☐	✓	☐

2. Do you think these foods are typically high or low in salt? (tick one box per food)

	High in salt	Low in salt	Not Sure
Breakfast cereals	✓	☐	☐
Frozen vegetables	☐	✓	☐
Bread	✓	☐	☐
Baked beans	✓	☐	☐
Red meat	☐	✓	☐
Canned soup	✓	☐	☐

3. Do you think these foods are typically high or low in fibre? (tick one box per food)

	High in fibre	Low in fibre	Not Sure
Oats	✓	☐	☐
Bananas	✓	☐	☐
White rice	☐	✓	☐
Eggs	☐	✓	☐
Potatoes with skin	✓	☐	☐
Pasta	☐	✓	☐

4. Do you think these foods are a good source of protein? (tick one box per food)

	Good source of protein	Not a good source of protein	Not sure
Poultry	✓	☐	☐
Cheese	✓	☐	☐
Fruit	☐	✓	☐
Baked beans	✓	☐	☐
Butter	☐	✓	☐
Nuts	✓	☐	☐

5. Which of the following foods do experts count as starchy foods? (tick one box per food)

	Starchy food	Not a starchy food	Not sure
Cheese	☐	✓	☐
Pasta	✓	☐	☐
Potatoes	✓	☐	☐
Nuts	☐	✓	☐
Plantains	✓	☐	☐

6. Which is the main type of fat present in each of these foods? (tick one box per food)

	Polyunsaturated fat	Monounsat-urated fat	Saturated fat	Cholesterol	Not sure
Olive oil	☐	✓	☐	☐	☐
Butter	☐	☐	✓	☐	☐
Sunflower oil	✓	☐	☐	☐	☐
Eggs	☐	☐	☐	✓	☐

7. Which of these foods has the most trans-fat? (tick one)

Biscuits, cakes and pastries	✓
Fish	☐
Rapeseed oil	☐
Eggs	☐
Not sure	☐

8. The amount of calcium in a glass of whole milk compared to a glass of skimmed milk is: (tick one)

About the same	✓
Much higher	☐
Much lower	☐
Not sure	☐

9. Which one of the following nutrients has the most calories for the same weight of food? (tick one)

Sugar	☐
Starchy	☐
Fibre/roughage	☐
Fat	✓
Not sure	☐

10. Compared to minimally processed foods, processed foods are: (tick one)

Higher in calories	✓
Higher in fibre	☐
Lower in salt	☐
Not sure	☐

Section 3: Knowledge of healthy food choices

1. If a person wanted to buy a yogurt at the supermarket, which would have the least sugar/sweetener? (tick one)

0% fat cherry yogurt ☐
Natural yogurt ✓
Creamy fruit yogurt ☐
Not sure ☐

2. If a person wanted soup in a restaurant or cafe, which one would be the lowest fat option? (tick one)

Mushroom risotto soup (field mushrooms, porcini mushrooms, arborio rice, butter, cream, parsley and cracked black pepper) ☐

Carrot butternut and spice soup (carrot , butternut squash, sweet potato, cumin, red chillies, coriander seeds and lemon) ✓

Cream of chicken soup (British chicken, onions, carrots, celery, potatoes, garlic, sage, wheat flour, double cream) ☐

Not sure ☐

3. Which would be the healthiest and most balanced choice for a main meal in a restaurant? (tick one)

Roast turkey, mashed potatoes and vegetables ✓
Beef, Yorkshire pudding and roast potatoes ☐
Fish and chips served with peas and tartar sauce ☐
Not sure ☐

4. Which would be the healthiest and most balanced sandwich lunch? (tick one)

Ham sandwich + fruit + blueberry muffin + fruit juice ☐
Tuna salad sandwich + fruit + low fat yogurt + water ✓
Egg salad sandwich + crisps + low fat yogurt + water ☐
Not sure ☐

5. Which of these foods would be the healthiest choice for a pudding? (tick one)

Berry sorbet ✓
Apple and blackberry pie ☐
Lemon cheesecake ☐
Carrot cake with cream cheese topping ☐
Not sure ☐

6. Which of these combinations of vegetables in a salad would give the greatest variety of vitamins and antioxidants? (tick one)

Lettuce, green peppers and cabbage ☐
Broccoli, carrot and tomatoes ✓
Red peppers, tomatoes and lettuce ☐
Not sure ☐

7. If a person wanted to reduce the amount of fat in their diet, but didn't want to give up chips, which of the following foods would be the best choice? (tick one)

Thick cut chips ✓
Thin cut chips ☐
Crinkle cut chips ☐
Not sure ☐

8. One healthy way to add flavour to food without adding extra fat or salt is to add: (tick one)

Coconut milk ☐
Herbs ✓
Soya sauce ☐
Not sure ☐

9. Which of the following cooking methods requires fat to be added? (tick one)

Grilling ☐
Steaming ☐
Baking ☐
Sautéing ✓
Not sure ☐

10. Traffic lights are often used on nutrition labelling, what would amber mean for the fat content of a food? (tick one)

Low fat ☐
Medium fat ✓
High in fat ☐
Not sure ☐

11. "Light" foods (or Diet foods) are always good options because they are low in calories. (tick one)

Agree ☐
Disagree ✓
Not sure ☐

The following questions are related to food labels:

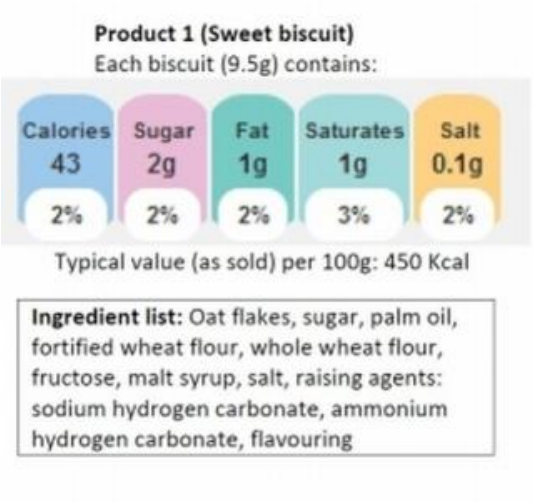

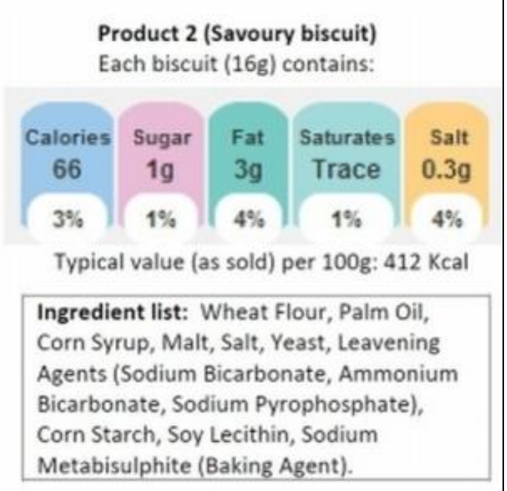

12. Looking at products 1 and 2, which one has the most calories (kcal) per 100 grams (tick one)

Product 1	✓
Product 2	☐
Both have the same quantity	☐
Not sure	☐

13. Looking at product 1, what are the sources of sugar in the ingredient list? (tick one)

Sugar and malt syrup	☐
Sugar, fructose and lecithin	☐
Sugar, fructose and malt syrup	✓
Not sure	☐

Section 4: Knowledge of diet, disease and weight associations

1. Which of these diseases is related to a low intake of fibre? (tick one)

Bowel disorders ✓
Anaemia ☐
Tooth decay ☐
Not sure ☐

2. Which of these diseases is related to how much sugar people eat? (tick one)

High blood pressure ☐
Tooth decay ✓
Anaemia ☐
Not sure ☐

3. Which of these diseases is related to how much salt (or sodium) people eat? (tick one)

Hypothyroidism ☐
Diabetes ☐
High blood pressure ✓
Not sure ☐

4. Which of these options do experts recommend to reduce the chances of getting cancer? (tick one)

Drinking alcohol regularly ☐
Eating less red meat ✓
Avoiding additives in food ☐
Not sure ☐

5. Which of these options do experts recommend to prevent heart disease? (tick one)

Taking nutritional supplements ☐
Eating less oily fish ☐
Eating less trans-fats ✓
Not sure ☐

6. Which of these options do experts recommend to prevent diabetes? (tick one)

Eating less refined foods ✓
Drinking more fruit juice ☐
Eating more processed meat ☐
Not sure ☐

7. Which one of these foods is more likely to raise people's blood cholesterol? (tick one)

Eggs ☐
Vegetable oils ☐
Animal fat ✓
Not sure ☐

8. Which one of these foods is classified as having a high Glycaemic Index (Glycaemic Index is a measure of the impact of a food on blood sugar levels, thus a high Glycaemic Index means a greater rise in blood sugar after eating)? (tick one)

Wholegrain cereals ☐
white bread ✓
Fruit and vegetables ☐
Not sure ☐

9. To maintain a healthy weight people should cut fat out completely. (tick one)

Agree ☐
Disagree ✓
Not sure ☐

10. To maintain a healthy weight people should eat a high protein diet. (tick one)

Agree ☐
Disagree ✓
Not sure ☐

11. Eating bread always causes weight gain. (tick one)

Agree ☐
Disagree ✓
Not Sure ☐

12. Fibre can decrease the chances of gaining weight. (tick one)

Agree ✓
Disagree ☐
Not sure ☐

13. Which of these options can help people to maintain a healthy weight? (answer each one)

	Yes	No	Not sure
Not eating while watching TV	✓	☐	☐
Reading food labels	✓	☐	☐
Taking nutritional supplements	☐	✓	☐
Monitoring their eating	✓	☐	☐
Monitoring their weight	✓	☐	☐
Grazing throughout the day	☐	✓	☐

14. If someone has a Body Mass Index (BMI) of 23kg/m^2, what would their weight status be? (tick one)

Underweight	☐
Normal weight	✓
Overweight	☐
Obese	☐
Not sure	☐

15. If someone has a Body Mass Index (BMI) of 31kg/m^2, what would their weight status be? (tick one)

Underweight	☐
Normal weight	☐
Overweight	☐
Obese	✓
Not sure	☐

Look at the body shapes below:

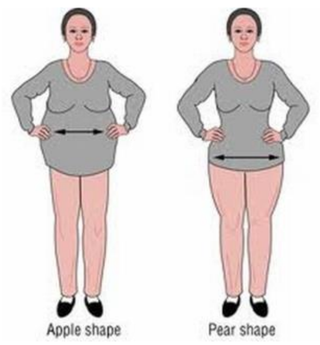

16. Which of these body shapes increases the risk of cardiovascular disease (Cardiovascular disease is a general term that describes a disease of the heart of blood vessels, for example, angina, heart attack, heart failure, congenital heart disease and stroke)? (tick one)

Apple shape	✓
Pear shape	☐
Not sure	☐
